# Hsa_circ_0134111 promotes osteoarthritis progression by regulating miR-224-5p/CCL1 interaction

**DOI:** 10.18632/aging.203420

**Published:** 2021-08-19

**Authors:** Yongbao Liu, Yanxiu Zhang

**Affiliations:** 1 Department of Imaging, First People’s Hospital of Lianyungang City, Lianyungang 222000, Jiangsu Province, China

**Keywords:** osteoarthritis, chondrocytes, hsa_circ_0134111, miR-224-5p, proliferation

## Abstract

Mechanical, metabolic, inflammatory, and immune factors contribute to the development of osteoarthritis (OA), a joint disease characterized by cartilage destruction. The circular RNA (circRNA) hsa_circ_0134111 is upregulated in the cartilage of OA patients; however, its potential role in OA pathogenesis and progression remains unexplored. In this study, the effects of hsa_circ_0134111 knockdown were evaluated in primary human chondrocytes treated with IL-1β to simulate OA, as well as in a rat model of OA. Hsa_circ_0134111 expression was upregulated in IL-1β-stimulated chondrocytes. CCK-8 and flow cytometry assays showed that hsa_circ_0134111 knockdown reversed IL-1β-induced cell decline by inhibiting apoptosis. Following prediction analysis of circRNA and miRNA targets, dual-luciferase reporter and silencing/overexpression assays suggested that a regulatory network composed of hsa_circ_0134111, miR-224-5p, and CCL1 modulates IL-1β-mediated OA-like effects in chondrocytes. Accordingly, CCL1 overexpression abrogated the prosurvival effects of hsa_circ_0134111 knockdown *in vitro*. Moreover, hsa_circ_0134111 silencing *in vivo* alleviated cartilage destruction in an OA rat model, decreased IL-6 and TNF-α levels in synovial fluid, and downregulated CCL1 expression in the affected joints. These results suggest that hsa_circ_0134111 contributes to OA development by binding to miR-224-5p, thereby releasing the inhibition that miR-224-5p exerts over CCL1.

## INTRODUCTION

Osteoarthritis (OA) is one of the most common joint diseases, characterized by degenerative changes in the articular cartilage that lead to aseptic inflammation [1]. The prevalence of OA increases with age and causative factors include environmental and lifestyle conditions, such as obesity and physical inactivity, joint wear and tear product of heavy physical work or activity, and trauma [2]. However, since the etiology and pathogenesis of OA are still unclear, treatment options for OA are still limited [3]. OA entails a large burden for public health systems and is associated with chronic pain and decreased life quality in those affected. Thus, discovery of novel therapies for the treatment of OA is of great importance.

Circular RNAs (circRNAs) comprise a class of non-coding RNAs (ncRNAs) with a closed circular structure that is widely represented in the eukaryotic transcriptome [4]. Different circRNAs play important functions in a wide variety of cells and contribute to various diseases by regulating gene expression at the post-transcriptional level [5]. Literature reports have shown that circRNAs are involved in the pathogenesis of OA [6, 7]. Zhou et al. suggested that circRNA 33186 is involved in the development of OA through sponging miR-127-5pECM to influence catabolism, proliferation, and apoptosis [6]. In turn, Wang et al. suggested a diagnostic value for hsa_circ_0032131_CBC1 in OA pathogenesis and identified hsa_circ_0134111 as a significantly upregulated ncRNA in OA tissues [7, 8]. In addition, Wu et al. reported that the importance of hsa_circ_0134111 in OA by regulating miR-515-5p [9].

In the current study, we explored the role of hsa_circ_0134111 in the occurrence and development of OA and unveiled its participation in a circRNA/miRNA/mRNA network involving hsa_circ_0134111/miR-224-5p/CCL1 in human chondrocytes. Interleukin-1β (IL-1β) is an important mediator of inflammatory response [10]. It is involved in a variety of cellular activities, including cell proliferation, differentiation, and apoptosis [11]. IL-1β is reported to be a key factor in inducing cartilage damage [12]. IL-1β acts on chondrocytes, interferes with the transformation of extracellular matrix, accelerates the degradation of chondrocyte matrix, and induces chondrocyte apoptosis [13]. Therefore, human primary chondrocytes were treated with IL-1β to mimic OA status *in vitro*. We hope that this study will further our understanding of the complex mechanisms governing OA progression and stimulate the search for new treatments.

## RESULTS

### Hsa_circ_0134111 knockdown prevents IL-1β-induced growth inhibition in chondrocytes

**Figure 1:**
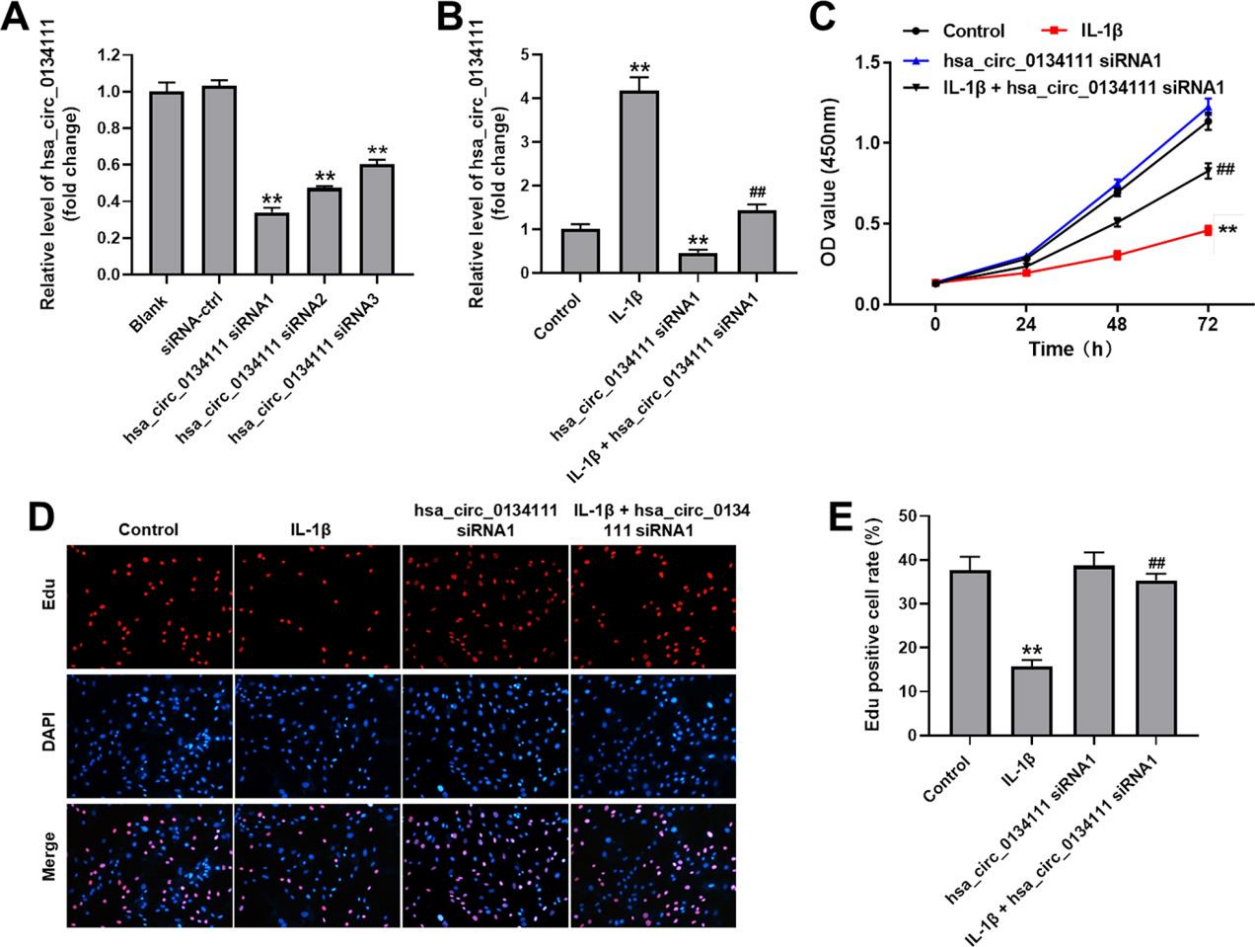
**Hsa_circ_0134111 knockdown prevents IL-1β-induced growth inhibition in chondrocytes.** (**A**) Chondrocytes were treated with hsa_circ_0134111 siRNA-ctrl, hsa_circ_0134111 siRNA1, hsa_circ_0134111 siRNA2, or hsa_circ_0134111 siRNA3 and RT-qPCR was performed to measure knockdown efficiency. (**B**) Chondrocytes were treated with IL-1β and hsa_circ_0134111 siRNA1, alone or in combination, and RT-qPCR was performed to measure hsa_circ_0134111 levels in cells. (**C**) Results of the CCK-8 assay, used to detect cell viability. (**D**, **E**) EdU and DAPI staining was used to measure chondrocyte proliferation. ^**^P<0.01 compared with control; ^##^P<0.01 compared with hsa_circ_0134111 siRNA1.

RT-qPCR was first performed in cultured human primary chondrocytes to evaluate both endogenous hsa_circ_0134111 levels and the effect on hsa_circ_0134111 expression of 3 different hsa_circ_0134111 siRNAs. As shown in Figure 1A, compared with control (NC) siRNA, all 3 hsa_circ_0134111 siRNAs obviously downregulated hsa_circ_0134111 expression in chondrocytes. Since hsa_circ_0134111 siRNA1 exhibited the highest inhibitory effect, this vector was selected for subsequent experiments. To mimic OA status *in vitro*, human primary chondrocytes were treated with IL-1β. RT-qPCR assays showed that IL-1β markedly increased the expression of hsa_circ_0134111 in chondrocytes, and this effect was completely reversed by hsa_circ_0134111 siRNA1 (Figure 1B). Next, the CCK-8 assay was used to evaluate cell viability. Results indicated that IL-1β significantly decreased cell viability, and this phenomenon was notably reversed by hsa_circ_0134111 siRNA1 (Figure 1C). To complement these findings, chondrocyte proliferation was examined using EdU staining. Consistent with the above data, IL-1β significantly inhibited cell proliferation, and this effect was reversed by pre-treatment with hsa_circ_0134111-1 siRNA1 (Figure 1D, 1E). These data indicate that IL-1β exposure upregulates hsa_circ_0134111-1 expression, resulting in inhibition of human chondrocyte growth.

### Hsa_circ_0134111 knockdown attenuates IL-1β-induced apoptosis, aggrecan downregulation, and MMP13 expression in chondrocytes

**Figure 2:**
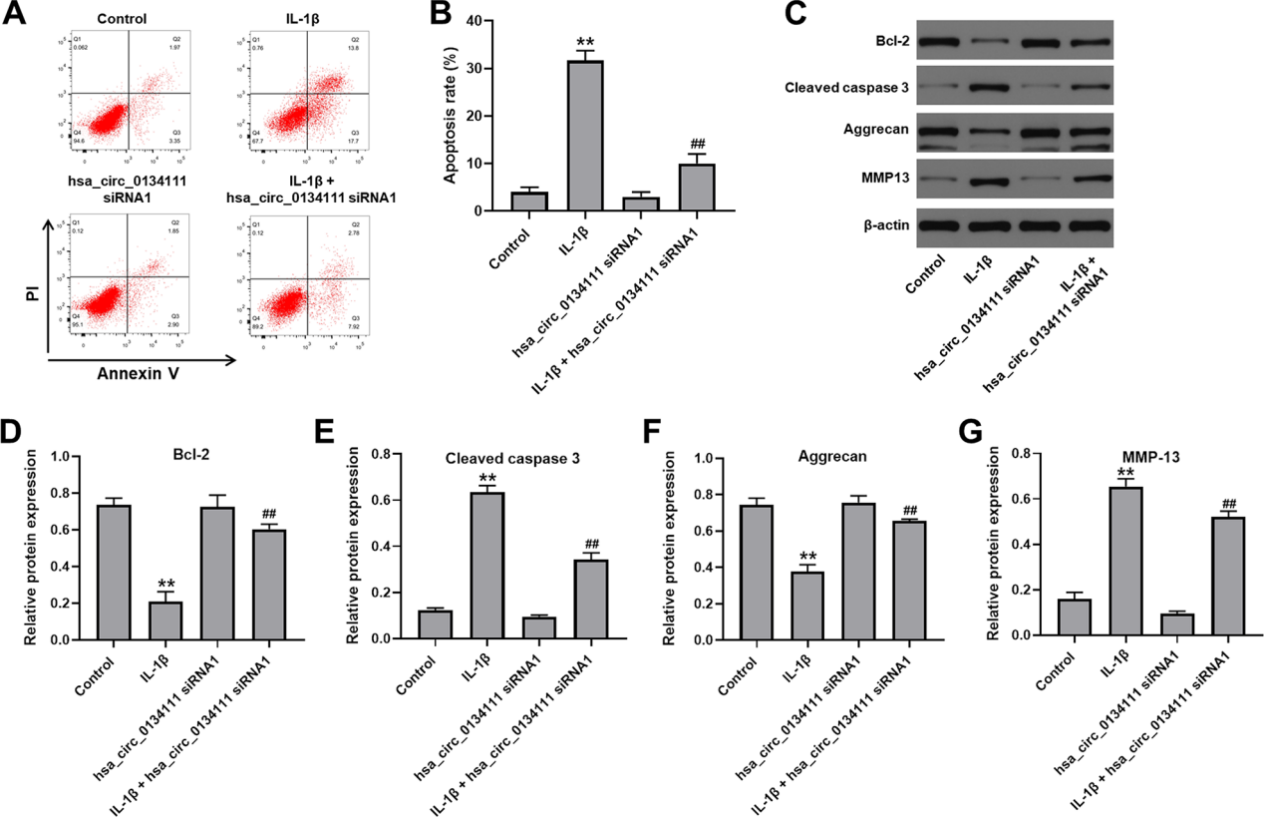
**Hsa_circ_0134111 knockdown alleviates IL-1β-induced apoptosis in chondrocytes.** (**A**, **B**) Flow cytometric analysis of apoptosis. (**C**–**G**) Representative western blot images and densitometric analysis of Bcl-2, cleaved caspase 3, aggrecan, and MMP13 expression in chondrocytes. ^**^P<0.01, compared with control; ^##^P<0.01, compared with hsa_circ_0134111 siRNA1.

We next used flow cytometry and western blotting assays to evaluate whether hsa_circ_0134111 knockdown can prevent IL-1β-induced apoptosis in chondrocytes. As indicated in Figure 2A, 2B, apoptosis was significantly induced upon exposure to IL-1β, whereas hsa_circ_0134111 siRNA1 pre-treatment markedly attenuated this effect. The molecular basis of apoptosis inhibition following hsa_circ_0134111 suppression was then examined by measuring the expression of apoptosis-related proteins. Results indicated that IL-1β downregulated Bcl-2 and upregulated cleaved caspase 3 in cells; however, these changes were abrogated by hsa_circ_0134111 siRNA1 (Figure 2C–2E).

It has been reported that downregulation of aggrecan, the major proteoglycan in the articular cartilage, and upregulation of matrix metalloproteinase 13 (MMP13), a main cartilage-degrading enzyme, are important events in the early stage of OA [14–18]. Western blotting showed that IL-1β significantly decreased the expression of aggrecan and increased the expression of MMP13 in cultured chondrocytes. Notably, these phenomena were largely prevented after transduction of hsa_circ_0134111 siRNA1 (Figure 2C, 2F, 2G). These data suggest that hsa_circ_0134111 expression in chondrocytes contributes to both apoptosis and proteoglycan degradation under simulated OA conditions.

### MiR-224-5p is targeted and negatively regulated by hsa_circ_0134111

**Figure 3:**
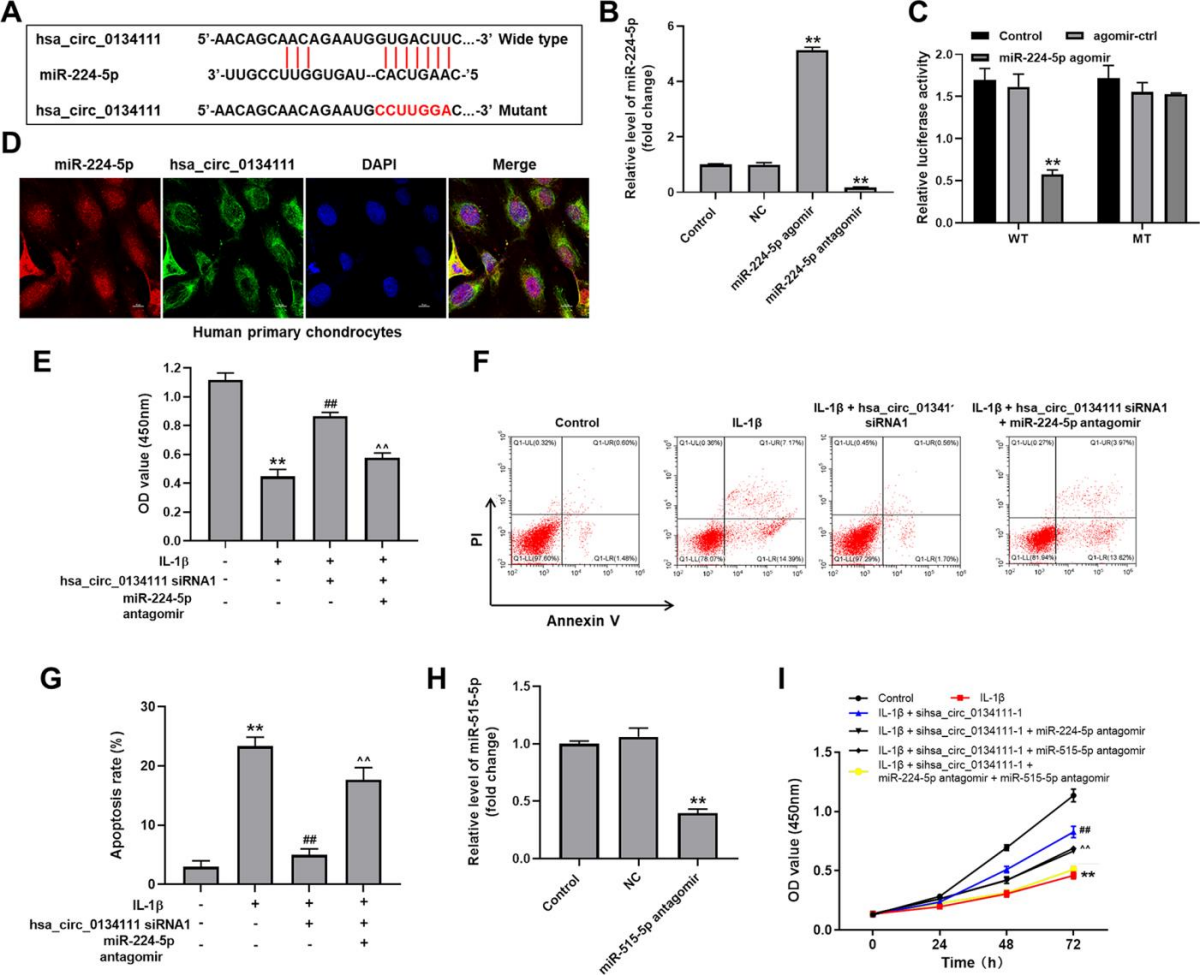
**MiR-224-5p is targeted and negatively regulated by hsa_circ_0134111.** (**A**) Diagram showing binding sites for miR-224-5p in the hsa_circ_0134111 sequence and nucleotide sequence of the mutant hsa_circ_0134111 construct. (**B**) Chondrocytes were treated with miR-224-5p NC, miR-224-5p agomir, or miR-224-5p antagomir and RT-qPCR was performed to measure miR-224-5p levels in cells. (**C**) A dual-luciferase reporter assay was used to evaluate binding of miR-224-5p to hsa_circ_0134111. (**D**) FISH experiment was performed to detect the co-localization of hsa_circ_0134111 and miR-224-5p in human primary chondrocytes. (**E**) CCK-8 assay was used to detect cell viability. (**F**, **G**) Flow cytometric analysis of apoptosis. (**H**) Chondrocytes were treated with miR-515-5p NC or miR-515-5p antagomir and RT-qPCR was performed to measure miR-515-5p levels in cells. (**I**) CCK-8 assay was performed to detect cell viability. ^**^P<0.01, compared with control; ^##^P<0.01, compared with IL-1β. ^^^^P<0.01, compared with IL-1β + hsa_circ_0134111 siRNA1.

To study the mechanism by which hsa_circ_0134111 potentially regulates the occurrence and development of OA, the online bioinformatics database CircInteractome (https://circinteractome.nia.nih.gov/) was interrogated to predict downstream targets of hsa_circ_0134111. This search identified miR-1238, miR-1248, miR-1307, miR-224-5p, and miR-578 as candidate targets. Among these miRNAs, miR-224-5p was reported to be closely related to the pathogenesis of OA [8, 19, 20]. Therefore, we focused on exploring the relationship between hsa_circ_0134111 and miR-224-5p. A diagram depicting potential binding sites between hsa_circ_0134111 and miR-224-5p is shown in Figure 3A. After artificially increasing and suppressing miR-224-5p expression by transfecting chondrocytes with miR-224-5p agomir or antagomir constructs, respectively (Figure 3B), a dual luciferase assay was conducted to verify the hsa_circ_0134111/miR-224-5p interaction. Results indicated that the luciferase activity of the hsa_circ_0134111 wild type (WT) reporter plasmid, unlike that of the control mutant (MT) sequence, was inhibited by miR-224-5p agomir transfection (Figure 3C). In contrast, transfection with miR-224-5p negative control (NC) did not affect hsa_circ_0134111-associated luciferase activity. In addition, the result of FISH exhibited the co-localization of hsa_circ_0134111 and miR-224-5p in human primary chondrocytes (Figure 3D). Moreover, CCK-8 assay showed that IL-1β significantly decreased cell viability, and this phenomenon was notably reversed by hsa_circ_0134111 siRNA1, and the added effect of hsa_circ_0134111 siRNA1 on cell viability was abolished by miR-224-5p antagomir (Figure 3E). Expectantly, IL-1β remarkably induced apoptosis, and this phenomenon was notably reversed by hsa_circ_0134111 siRNA1, and the inhibitory effect of hsa_circ_0134111 siRNA1 on apoptosis was abolished by miR-224-5p antagomir (Figure 3F, 3G).

Furthermore, Wu et al. reported that hsa_circ_0134111 was overexpressed in IL-1β-induced chondrocytes [9]. In addition, we examined the effects of miR-224-5p antagomir or/and miR-515-5p antagomir on IL-1β-induced human primary chondrocytes. IL-1β significantly decreased cell viability, and this phenomenon was notably reversed by hsa_circ_0134111 siRNA1, however, the reversal effect of hsa_circ_0134111 siRNA1 on cell viability was abolished by miR-224-5p antagomir or miR-515-5p antagomir. Just as we expected, on this basis, the combination of miR-224-5p antagomir and miR-515-5p antagomir inhibited cell activity more significantly than when they were used alone (Figure 3H, 3I). These data suggest that miR-224-5p is targeted and negatively regulated by hsa_circ_0134111. In addition, hsa_circ_0134111 mediates the OA progression by modulating miR-224-5p and miR-515-5p pathways in a synergistic way.

### MiR-224-5p negatively regulates CCL1 expression in human chondrocytes

**Figure 4:**
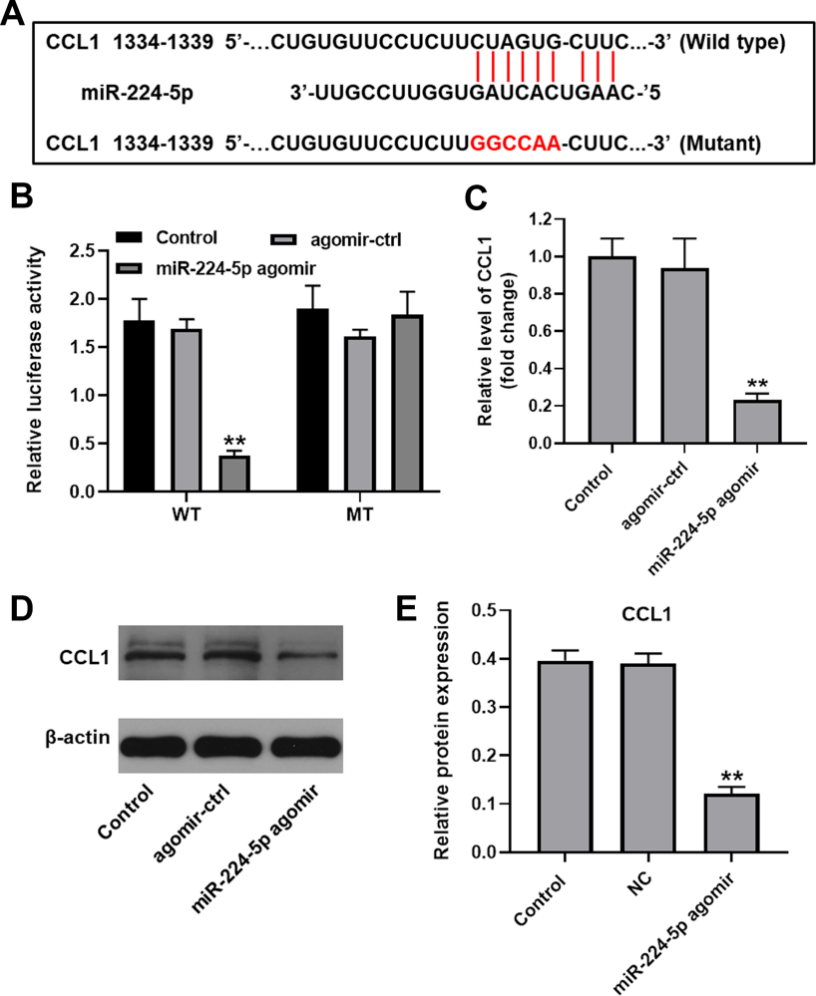
**CCL1 is targeted and negatively regulated by miR-224-5p.** (**A**) Diagram showing binding sites for miR-224-5p in the CCL1 mRNA and nucleotide sequence of the mutant CCL1 3’-UTR. (**B**) A dual-luciferase reporter assay was used to confirm the binding of CCL1 to miR-224-5p. (**C**) Chondrocytes were treated with miR-224-5p agomir-ctrl or miR-224-5p agomir and RT-qPCR was performed to measure CCL1 levels in cells. (**D**, **E**) Western blot was used to detect the level of CCL1. ^**^P<0.01, compared with control.

To identify putative target genes of miR-224-5p, three online bioinformatics databases (TargetScan, miRDB, and miRWalk) were accessed. Among the genes retrieved, we selected chemokine ligand 1 (CCL1) based on its reported role in OA progression [21] (Figure 4A). Suggesting that miR-224-5p binds specifically to CCL1, dual luciferase assays in cultured chondrocytes demonstrated effective inhibition of the activity of the CCL1 reporter containing a WT, but not a control MT, 3’-UTR upon expression of miR-224-5p agomir (Figure 4B). This finding was further supported by RT-qPCR data, which showed that transfection with miR-224-5p agomir significantly decreased the expression of CCL1 (Figure 4C). In addition, western blot assays showed that miR-224-5p agomir significantly decreased the expression of CCL1, compared with the control group (Figure 4D, 4E). These results indicate that CCL1 is negatively regulated by direct binding of miR-224-5p.

### Hsa_circ_0134111 knockdown counteracts IL-1β-induced chondrocyte death by regulating the miR-224-5p/CCL1 axis

**Figure 5:**
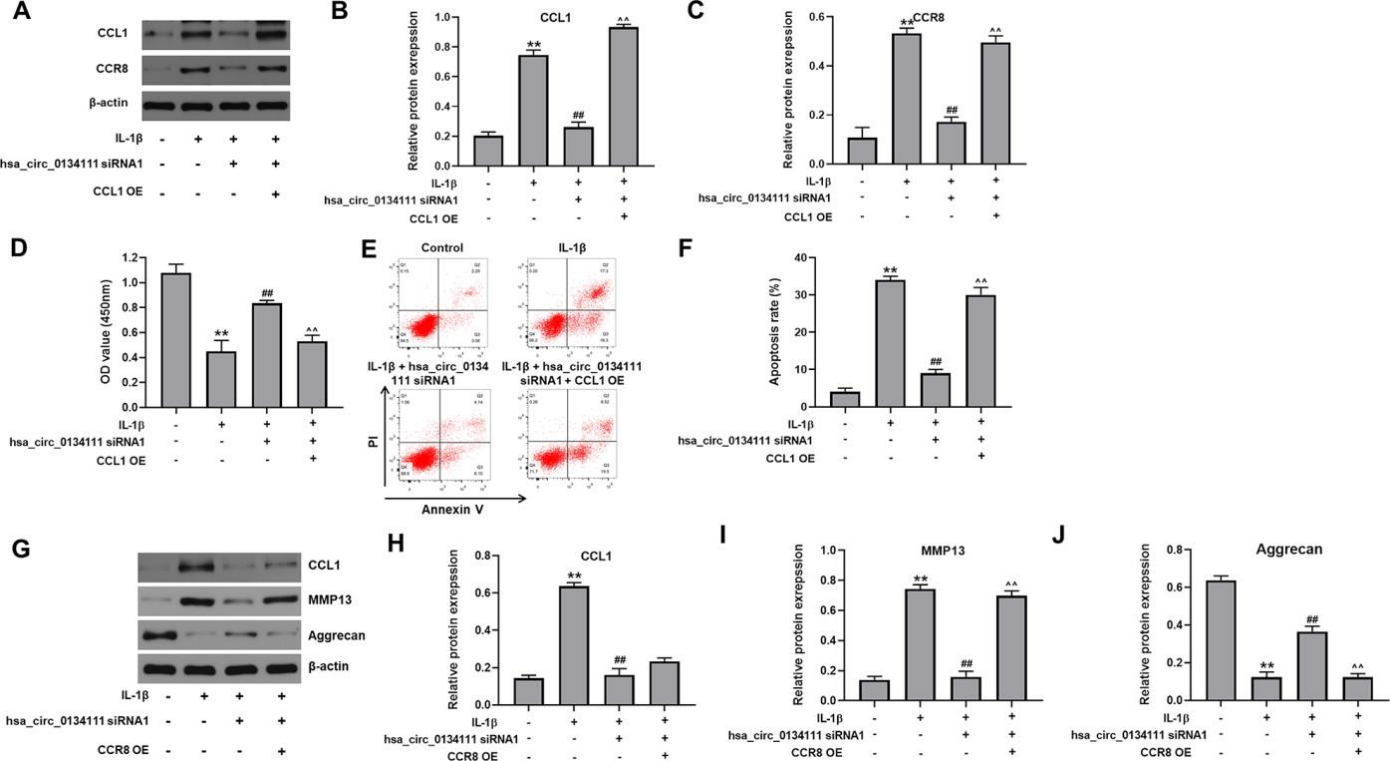
**Hsa_circ_0134111 knockdown counteracts IL-1β-induced chondrocyte death by regulating the miR-224-5p/CCL1 axis.** (**A**–**C**) Representative western blot images and densitometric analysis of CCL1 and CCR8 expression in cultured chondrocytes. (**D**) Cell viability (CCK-8) assay results. (**E**, **F**) Flow cytometric analysis of apoptosis. (**G**–**J**) Western blots results. ^**^P<0.01, compared with control; ^##^P<0.01, compared with IL-1β. ^^^^P<0.01, compared with IL-1β + hsa_circ_0134111 siRNA1.

To further explore the regulatory action of hsa_circ_0134111 on the miR-224-5p/CCL1 interaction and OA progression, we first assessed the expression of CCR8, the CCL1 receptor [18], after lentivirus-mediated overexpression (OE) of CCL1 (CCL1 OE). Western blot assays revealed that IL-1β exposure significantly increased the expression of CCL1 and CCR8 in chondrocytes, and these changes were markedly reversed by hsa_circ_0134111 siRNA1. However, the inhibitory effect of hsa_circ_0134111 siRNA1 on both CCL1 and CCR8 expression was abolished by CCL1 OE (Figure 5A–5C). Moreover, in IL-1β-treated cells, the rescuing effects of hsa_circ_0134111 siRNA1 on both cell viability and apoptosis were also largely negated by CCL1 OE (Figure 5D–5F).

Furthermore, western blotting showed that IL-1β notably increased the expression of CCL1, and this increase was reversed by hsa_circ_0134111 siRNA1; and CCR8 OE has no obvious effect on this phenomenon (Figure 5G, 5H). In addition, IL-1β remarkably increased the expression of MMP13 and decreased the expression of aggrecan, while these phenomena were notably reserved by hsa_circ_0134111 siRNA1. However, the effect of hsa_circ_0134111 siRNA1 on both MMP13 and aggrecan expression was abolished by CCR8 OE (Figure 5G, 5I, 5J). These results suggest that IL-1β-induced hsa_circ_0134111 expression promotes OA, at least in part, by releasing the inhibitory effect of miR-224-5p on its target gene CCL1.

### Hsa_circ_0134111 knockdown alleviates symptoms of OA *in vivo*

**Figure 6:**
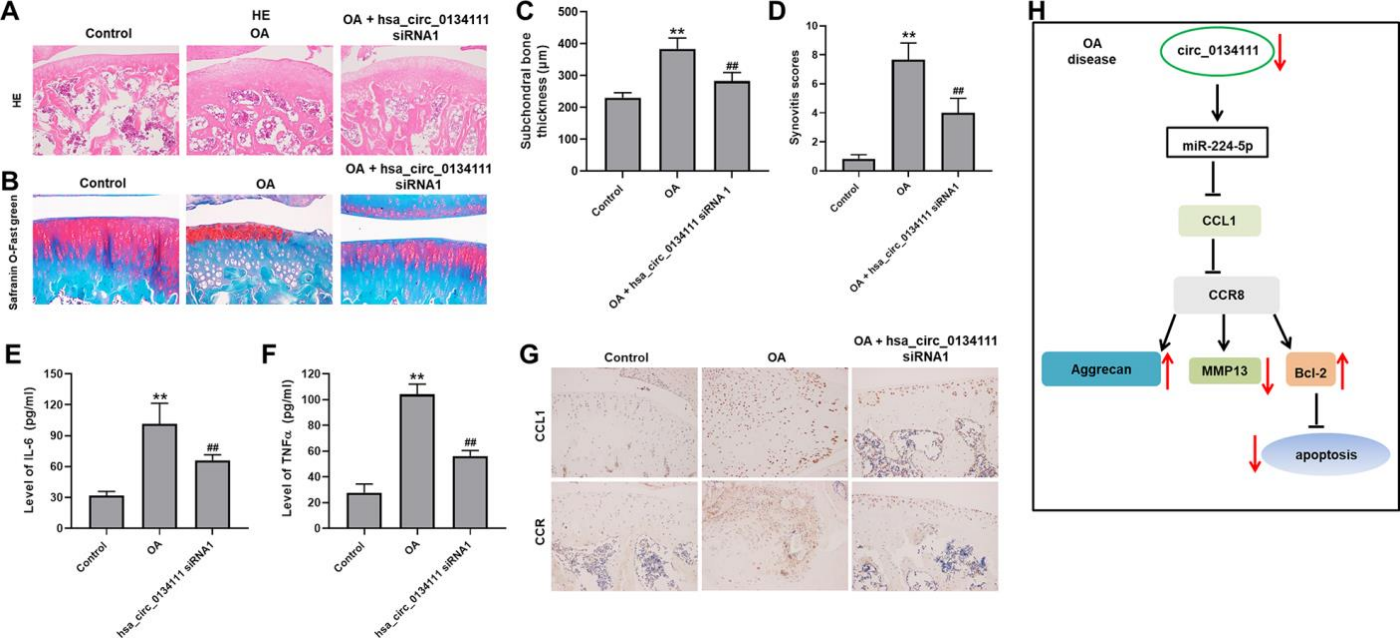
**Hsa_circ_0134111 knockdown alleviates the symptoms of OA *in vivo*.** Representative images of knee articular tissue stained with (**A**) H&E and (**B**) Safranin O/Fast Green. (**C**) Subchondral bone thickness measurements. (**D**) Synovitis scores for knee joints in the different experimental treatments. (**E**, **F**) ELISA-based measurements of IL-6 and TNF-α levels in knee synovia. (**G**) Representative IHC images showing the expression of CCL1 and CCR8 in knee joint tissue. (**H**) A graphical abstract of this study. ^**^P<0.01, compared with control; ^##^P<0.01, compared with OA.

To further confirm the role of hsa_circ_0134111 on the pathogenesis of OA, an OA model was established in rats by transecting the right knee’s medial meniscotibial ligament. After surgery, a group of rats received over 8 weeks periodic intra articular injections of hsa_circ_0134111 siRNA1. Following sacrifice, Safranin O/Fast Green and H&E staining showed substantial damage to the articular cartilage in the OA group. In contrast, a marked attenuation of tissue damage was observed in rats administered hsa_circ_0134111 siRNA1 (Figure 6A, 6B). Compared with the control group (sham surgery) subchondral bone thickness was obviously increased in the OA group, and this increase was reversed by hsa_circ_0134111 siRNA1 treatment (Figure 6C). Consistent with these data, the synovitis scores of the OA group were dramatically higher than those of the control group, but significantly reduced in the OA + hsa_circ_0134111 siRNA1 group (Figure 6D). To investigate the inflammatory status of the synovial fluid, ELISA was used to examine levels of inflammation-related factors, namely IL-6 and TNF-α. Relative to control samples, both IL-6 and TNF-α contents were markedly increased in the OA group, while hsa_circ_0134111 siRNA1 treatment significantly inhibited such increase (Figure 6E, 6F). As expected, the expression of CCL1 and CCR8 was significantly increased in the OA group compared with the control group; however, the expression of both proteins was notably reduced by hsa_circ_0134111 siRNA1 treatment (Figure 6G). In summary, these data showed that knockdown of hsa_circ_0134111 alleviates the symptoms of OA *in vivo*. Collectively, hsa_circ_0134111 inhibit IL-1β-induced apoptosis of human primary chondrocytes by regulating hsa_circ_0134111/miR 224 5p/CCL1/CCR8 pathways (Figure 6H).

## DISCUSSION

The present study highlights a novel mechanism by which hsa_circ_0134111 potentially regulates the occurrence and development of OA. We found that IL-1β, a pro-inflammatory cytokine that acts as the main driver of cartilage degradation during OA [22], markedly increased the expression of hsa_circ_0134111 in human chondrocytes. Knockdown of hsa_circ_0134111 in these cells significantly attenuated IL-1β-induced growth inhibition and apoptosis, which is consistent with the observed reversal of the downregulation of Bcl-2 and the upregulation of cleaved caspase 3 induced by IL-1β.

Several studies suggested that circRNAs play an important role in the development of OA [4, 5]. Huang et al. showed that circRNA_0092516 impairs proliferation and promotes apoptosis in IL-1β-stimulated chondrocytes by regulating the miR-337-3p/PTEN axis [4]. Zhu et al. reported that silencing of circGCN1L1 (hsa_circ_0000448) alleviates the symptoms of OA by regulating miR-330-3p and the TNF-α gene in synoviocytes [5]. Consistent with role of hsa_circ_0134111 demonstrated in our study, Zhou et al. found that IL-1β significantly increased the expression of circRNA.33186 in chondrocytes, and its silencing alleviated IL-1β-induced apoptosis [6].

Accumulating evidence shows that aggrecan downregulation and MMP13 upregulation are important events in the early stage of OA [14–18]. After joint fracture, increments is the expression of MMPs and in the degradation of aggrecan are observed [16]. It has been reported that hsa_circ_0005105 can inhibit the expression of aggrecan and promote the expression of MMP-13 by sponging miR-26a [23]. Similarly, we showed that IL-1β decreases aggrecan synthesis and augments MMP13 production in chondrocytes, two phenomena that were largely attenuated by hsa_circ_0134111 knockdown.

Dysregulated expression of miR-224-5p [19] and CCL1 [24, 25] has been implicated in the development of OA. Using luciferase reporter assays, we showed that miR-224-5p directly targets both hsa_circ_0134111 and CCL1, thus defining a competing endogenous RNA (ceRNA) regulatory network operational in chondrocytes and potentially involved in the development of OA. In such regard, our data would suggest that joint inflammation triggers hsa_circ_0134111 expression in chondrocytes, which sponges miR-224-5p to allow translation of its target gene CCL1, thus promoting OA. This mechanism is supported by the fact that CCL1 overexpression abrogated the pro-survival effect of hsa_circ_0134111 in IL-1β-treated chondrocytes. Nevertheless, further research is needed to clarify the specific role of CCL1 in chondrocyte inflammation and apoptosis in the setting of OA.

Moreover, Wu et al. reported that hsa_circ_0134111 was overexpressed in IL-1β-induced chondrocytes [9]. Our results are consistent with this. In addition, we examined hsa_circ_0134111 mediates the OA progression by modulating miR-224-5p and multiple miRNA pathways in a synergistic way.

Lastly, our *in vivo* experiments in a rat model of OA demonstrated that hsa_circ_0134111 inhibition attenuated articular damage and reduced IL-6 and TNF-α levels in synovial fluid from affected joints. These findings suggest that knockdown of hsa_circ_0134111 alleviates the progression of OA *in vitro* and *in vivo* by regulating the miR-224-5p/CCL1 axis. Hsa_circ_0134111 might thus represent a new target for the treatment of OA.

## MATERIALS AND METHODS

### Cell culture

Human primary chondrocytes were purchased from Procell (Wuhan, Hubei, China) and 293T cells were provided by the American Type Culture Collection (Manassas, VA, USA). Cells were cultured in RPMI-1640 medium (Thermo Fisher Scientific, Waltham, MA, USA) containing 10% FBS (Thermo Fisher Scientific) and 2 mM glutamine (Sigma, St. Louis, MO, USA), at 37° C with 5% CO_2_. To mimic OA *in vitro*, chondrocytes were treated with IL-1β (10 ng/mL) for 24 h [2, 26].

### Transfection and transduction procedures

SiRNAs against hsa_circ_0134111 (siRNAs1-3), as well as control siRNA were purchased from RiboBio (Guangzhou, China). Helper packaging vectors (pLP/VSVG, pLP1, and pLP2; 1 μg/μL) were purchased from Invitrogen (Thermo Fisher Scientific). MiR-224-5p agomir NC, miR-224-5p agomir, and miR-224-5p antagomir were purchased from GenePharma (Shanghai, China). CCL1-expressing lentivirus and CCR8-expressing lentivirus was obtained from Thermo Fisher Scientific. 293T cells were transduced with hsa_circ_0134111 siRNA1 or with CCL1-expressing lentivirus using Lipofectamine^®^ 2000 (Thermo Fisher Scientific). After incubation at 37° C for 48 h, cell supernatants containing retroviral particles were collected by centrifugation and viral particles harvested by passing the supernatant through a 0.45 μm filter. Incorporation of siRNAs (10 μM) and miR-224-5p or miR-515-5p agomir/antagomir (10 nM) into chondrocytes was carried out with Lipofectamine^®^ 2000 (Thermo Fisher Scientific). Human primary chondrocytes were incubated with viral particles for 48 h at 37° C before downstream analyses. Quantitative reverse transcription PCR (RT-qPCR) was used to verify knockdown/upregulation efficiency for the different procedures.

### RT-qPCR

TRIzol^®^ reagent (Thermo Fisher Scientific) was used to isolate total RNA from chondrocytes. To synthesize first-strand cDNA, an EntiLink^™^ 1st Strand cDNA Synthesis Kit (ELK Biotechnology) was used. Then, RT-qPCR was performed on a StepOne™ real-time PCR instrument (Life Technologies) with EnTurbo^™^ SYBR Green PCR SuperMix Kit (ELK Biotechnology Co., Wuhan, China). Primer sequences were: β-actin, Forward: 5’-GTCCACCGCAAATGCTTCTA-3’, Reverse: 5’-TGCTGTCACCTTCACCGTTC-3’; hsa-circ-0134111, Forward: 5’-GAAAACAGATGAGGAGAAGGCC-3’, Reverse: 5’-CGTCTTTTTCTCAGCTTTGCC-3’; CCL1, Forward: 5’-CCAGATGTTGCTTCTCATTTGC-3’, Reverse: 5’-CAGGGCAGAAGGAATGGTGT-3’; U6, Forward: 5’-CTCGCTTCGGCAGCACAT-3’, Reverse: 5’-AACGCTTCACGAATTTGCGT-3’; hsa-miR-224-5p, Forward: 5’-CAAGTCACTAGTGGTTCCGTTTAG-3’, Reverse: 5’-CTCAACTGGTGTCGTGGAGTC-3’; miR-515-5p Forward: 5’-CGGGTTCTCCAAAAGAAAGCA-3’, Reverse: 5’-CAGCCACAAAAGAGCACAAT-3’. U6 was used as the internal control for hsa-miR-224-5p and miR-515-5p. The internal reference for the other genes was β-actin, and we used the 2^–ΔΔCT^ method for analysis of gene expression.

### Cell viability assay

The viability of chondrocytes was assessed through the CCK-8 assay. Chondrocytes (5×10^3^ per well) were cultured in 96-well plates. After experimental treatments, 10 μl of CCK-8 reagent (Beyotime, Shanghai, China) was added to each well for 2 h at 37° C. Optical density was obtained at 450 nm with a microplate reader (Bio-Rad, Hercules, CA, USA).

### Cell proliferation assay

The proliferation of chondrocytes was detected by EdU (5-ethynyl-2’-deoxyuridine) staining using an EdU Detection kit (RiboBio Biology, Guangzhou, China). Chondrocytes were incubated at 37° C with 50 μM EdU for 2 h followed by staining with the Apollo reaction cocktail for 30 min at 37° C in the dark. A fluorescence microscope (Olympus, Tokyo, Japan) was used to analyze EdU uptake.

### Cell apoptosis analysis

Annexin V-FITC Apoptosis Detection Kit (Beyotime) was used to detected cell apoptosis. Chondrocytes (5×10^4^ per well) were cultured in 6-well plates. Following experimental treatments, the cells were incubated with 5 μl Annexin V-FITC and 5 μl PI for 15 min at 4° C and apoptosis evaluated by flow cytometry (BD Biosciences, San Jose, CA, USA).

### Western blotting

Protein lysis buffer (Beyotime) was used to extract proteins from chondrocytes and their concentrations determined using a BCA protein assay kit (Aspen Biotechnology, Wuhan, China). SDS-PAGE (10%) was used to separate equal amounts of protein (40 μg) per sample. After transfer onto polyvinylidene fluoride (PVDF) membranes, TBST containing 5% skim milk was applied at room temperature for 1h. Then, the membranes were incubated with the following primary antibodies: anti-Aggrecan (1:1000; Cat. ab3778), anti-MMP13 (1:1000; Cat. ab51072), anti-Bcl-2 (1:1000; Cat. ab32124), anti-cleaved caspase 3 (1:1000; Cat. ab32042), anti-CCL1 (1:1000; Cat. ab280356), anti-CCR8 (1:1000; Cat. ab32399), and anti-β-actin (1:1000; Cat. ab8226) at 4° C overnight. Subsequently, HRP-conjugated corresponding secondary antibodies (Abcam; ab7356, 1:5000) were applied at room temperature for 1 h. All the antibodies were purchased from Abcam (Cambridge, MA, USA). Protein bands were detected using an enhanced chemiluminescence (ECL) kit (Thermo Fisher Scientific). β-actin served as a loading control.

### *In vivo* model of OA

A total of 15 female wistar rats (12-week-old) were purchased from the Animal Center of the Chinese Academy of Sciences (Shanghai, China). All animal procedures adhered to the recommendations of the Guide for the Care and Use of Laboratory Animals (National Institutes of Health, USA) [27]. Three experimental groups (n = 6 rats per group) were defined: 1) OA; 2) OA plus hsa_circ_0134111 siRNA1; and 3) Control (sham surgery). To establish an OA model *in vivo*, rats were anesthetized by intraperitoneal injection of 2% pentobarbital (40 mg/kg) and then the joint capsule of the right knee was incised medial to the patellar tendon. The medial meniscotibial ligament was then transected using microsurgical scissors as previously described [28]. Control rats received a sham procedure consisting of temporarily exposing the joint capsule of the knee, without further tissue manipulation. Twenty-four hours after surgery, rats in the OA + hsa_circ_0134111 siRNA1 group were injected with hsa_circ_0134111 siRNA1 (50 nm) into the right joint cavity twice a week. OA rats were received in turn saline injections. Eight weeks after surgery, the animals were sacrificed with CO_2_ and knee joint tissues collected for downstream analyses.

### Dual-luciferase reporter assay

Wild-type (WT) and mutant (MT) reporter vectors for hsa_circ_0134111 and CCL1 containing putative binding sites for miR-224-5p were constructed with Sangon Biotech (Shanghai, China) and cloned into pGL6 vectors (Beyotime). Chondrocytes were transfected over 48 h with hsa_circ_0134111/CCL1 3’-UTR-WT or hsa_circ_0134111/CCL1 3’-UTR-MT vectors, together with agomir-control or miR-224-5p agomir, using Lipofectamine^®^ 2000. A Dual-Luciferase Reporter Assay System (Beyotime) was used to detect firefly luciferase activity in chondrocyte lysates. Renilla luciferase activity served as transfection control.

### Fluorescence *in situ* hybridization analysis (FISH)

FISH experiment was performed to detect the co-localization of hsa_circ_0134111 and miR-224-5p in human primary chondrocytes. FITC-labeled miR-224-5p and Cy3-labeled hsa_circ_0134111 probes was provided by Biosense (Guangzhou, China). Fluorescent *In Situ* Hybridization Kit was used to hybridize. DAPI is used to stain the nucleus for 20 min. Finally, images were analyzed using a fluorescence microscope.

### Histopathological analysis

Safranin O and Fast Green staining was used for histological examination of joint tissue specimens. Morphological changes of cartilage and subchondral bone were assessed by observers blind to experimental treatment. The conditions of the medial femoral condyle and the medial tibial plateau were assessed using a synovitis scoring system [29]. Cartilage damage was further assessed by H&E staining.

### ELISA

The levels of IL-6 and TNF-α in synovial fluid were detected using specific ELISA kits. Interleukin-6 ELISA Kit and Tumor Necrosis Factor-α Assay Kit (Cat. H007-1-1 and H052-1) were provided by Nanjing Jiancheng Company (Nanjing, China).

### Immunohistochemistry

Osteoarticular tissues were fixed with 4% paraformaldehyde in PBS overnight, embedded in paraffin, and sliced into 5-μm-thick sections. Following deparaffinization and hydration, anti-CCL1 (1:1000; Cat. ab280356) and anti-CCR8 (1:1000; Cat. ab32399) antibodies were applied to the samples overnight at 4° C. DAB (3,3’-diaminobenzidine) was used to detect immunostaining. All the antibodies were purchased from Abcam (Cambridge, MA, USA).

### Statistical analysis

GraphPad Prism version 7.0 (GraphPad Software, La Jolla, CA, USA) was used for data analysis. Experiments were repeated at least three times and data are expressed as the mean ± SD. ANOVA and Tukey’s tests were used to compare differences between multiple groups. *P<0.05 was considered significant.

### Availability of data and materials

The datasets used and/or analyzed during the current study are available from the corresponding author on reasonable request.
